# Building a neurocognitive profile of suicidal risk in severe mental disorders

**DOI:** 10.1186/s12888-022-04240-3

**Published:** 2022-09-26

**Authors:** Anna Comparelli, Valentina Corigliano, Benedetta Montalbani, Adele Nardella, Antonella De Carolis, Lorenzo Stampatore, Paride Bargagna, Francesca Forcina, Dorian Lamis, Maurizio Pompili

**Affiliations:** 1 Azienda Ospedaliero-Universitaria Sant’Andrea, Sapienza University of Rome, Via di Grottarossa 1035, Rome, Italy; 2 Department of Psychiatry Roma 1, Rome, Italy; 3 Psychiatry Residency Training Program, Faculty of Medicine and Psychology, Sapienza University of Rome, Rome, Italy; 4 Department of Psychiatry and Substance Abuse, Modena, Italy; 5 Department of Neurosciences, Unit of Neurology, Mental Health and Sensory Organs, Sant’Andrea Hospital, Sapienza University, 00185 Rome, Italy; 6 Department of Psychiatry and Behavioral Sciences, Emory University School of Medicine, Atlanta, GA USA; 7 Suicide Prevention Centre, Department of Neurosciences, Mental Health and Sensory Organs, Sant’Andrea Hospital, Sapienza University, 00185 Rome, Italy

**Keywords:** Suicide ideation, Suicide attempt, Neurocognition, Social cognition, MATRICS Consensus Cognitive Battery

## Abstract

**Background:**

Research on the influence of neurocognitive factors on suicide risk, regardless of the diagnosis, is inconsistent. Recently, suicide risk studies propose applying a trans-diagnostic framework in line with the launch of the Research Domain Criteria Cognitive Systems model. In the present study, we highlight the extent of cognitive impairment using a standardized battery in a psychiatric sample stratified for different degrees of suicidal risk. We also differentiate in our sample various neurocognitive profiles associated with different levels of risk.

**Materials and methods:**

We divided a sample of 106 subjects into three groups stratified by suicide risk level: Suicide Attempt (SA), Suicidal Ideation (SI), Patient Controls (PC) and Healthy Controls (HC). We conducted a multivariate Analysis of Variance (MANOVA) for each cognitive domain measured through the standardized battery MATRICS Consensus Cognitive Battery (MCCB).

**Results:**

We found that the group of patients performed worse than the group of healthy controls on most domains; social cognition was impaired in the suicide risk groups compared both to HC and PC. Patients in the SA group performed worse than those in the SI group.

**Conclusion:**

Social cognition impairment may play a crucial role in suicidality among individuals diagnosed with serious mental illness as it is involved in both SI and SA; noteworthy, it is more compromised in the SA group fitting as a marker of risk severity.

## Introduction

Suicide is a significant public health concern with substantial individual and economic burdens. Every year, about 700,000 people die by suicide, with the global annual mortality rate estimated in 2017 by the World Health Organization to be 10.7 per 100,000 individuals, with variations across age groups and countries. Although the association of suicide risk with increased psychopathology functional impairment is well established among psychiatric patients and in the general population [1–3] current research on neurocognitive factors and suicide risk is inconsistent. In fact, some studies have described poorer neurocognitive performances associated with both suicidal ideation and attempt [4, 5], whereas, others [6, 7] found preserved cognitive functions in patients who attempted or died by suicide, compared to patients with suicide risk but without attempts. In the latter, researchers suggested that intact neurocognitive functions may facilitate planning suicidal behavior. However, most of the studies were composed of patients with a single diagnosis, particularly psychotic or mood disorders. Moreover, they did not utilize a standardized battery of tests, which limited the ability to compare and interpret data.

To overcome the limits imposed by the focus on neurocognitive features of suicidal risk in categorical disorders, some researchers have proposed to apply a dimensional approach to suicidality, in order to identify risk factors associated with suicide risk per se, regardless of diagnosis [8]. This perspective is in line with the more recent dimensional approach to psychopathological and behavior disorders [9].

With the aim of identifying cognitive commonalities of suicide risk across all psychiatric disorders, Huber et al. [8] conducted a systematic review of the literature examining cognitive deficits as a transdiagnostic risk factor for suicidality, especially regarding alterations in cognitive control. However, this study excluded data on neurocognitive domains that are critically important for suicide research such as social cognition. Moreover, Huber et al., do not differentiate between ideators and attempters, whereas a recent review [10] proposed that neurocognitive abilities might distinguish between attempters and non-attempters with regard to suicide risk.

Thus, given the heterogeneity of suicidal behavior along with the wide variety of cognitive deficits reported and the vast array of neurocognitive tests used in these studies, it is unclear which neurocognitive targets are most relevant to address using cognitive-based psychotherapeutic or cognitive remediation strategies.

Another question that remains open is whether individuals with suicide ideation exhibit distinct neurocognitive deficits that can be distinguished from those in suicide attempters.

Promisingly, the Measurement and Treatment Research to Improve Cognition in Schizophrenia (MATRICS) Consensus Cognitive Battery (MCCB) [11, 12] which has been validated for the assessment of neurocognition in schizophrenia [13–15], encompasses cognitive domains that are impacted by various psychiatric disorders [16–20].

The first aim of the present study was to compare a sample of psychiatric patients, stratified by different degree of suicidal risk with a group of healthy controls, in order to highlight the extent of their cognitive impairment on a standardized battery.

The second aim was to distinguish, in the context of severe mental disorders, various neurocognitive profiles associated with different suicidal risk.

With the delineated aims, we intend to study neurocognition as a transdiagnostic and trans-staging marker across the continuum of suicide risk from ideation to attempt.

Consistent with this perspective, we hypothesized that each suicidal risk condition would be associated with a specific neurocognitive profile, regardless of specific categorical diagnosis.

The clinical relevance of our study concerns the definition of target intervention for specific profiles in order to prevent suicide among individuals with severe mental illness.

## Materials and methods

### Subjects

We enrolled 106 subjects; 70 patients over the age of 18 years who were referred to our outpatient service and 36 healthy volunteers. Patients met a diagnosis of Bipolar and Related Disorders (19) or Cluster B Personality Disorders (16) or Schizophrenia Spectrum (28) and Other Psychotic Disorders (7) based on Structured Clinical Interview for DSM-5, Research Version SCID-5-RV [21]. The sample was divided into four groups: 1) patients with previous suicide attempts (SA) *(N* = *23)*, 2) patients who experienced only suicidal ideation (SI) *(N* = *20)*, 3) patients without history of suicidal ideation or suicidal behaviour (Patient Controls PC) *(N* = *27),* and 4) healthy controls (HC) (N = 36). Patients were clinically stable for a minimum of 3 months and were in treatment with medium doses of antipsychotics (doses equivalent to 13,3 mg/die Olanzapine), antidepressants (doses equivalent to 20 mg/die Fluoxetine), anxiolytics (doses equivalent to 4 mg/die Diazepam) or mood stabilizers (dose equivalent to 1000 mg/die valproate). Exclusion criteria for patients were (1) current or past comorbid diagnosis of autistic disorder or other pervasive developmental disorder, (2) history of severe head injury, (3) severe medical conditions or major neurological disorders, including mental retardation and dementia, which could prevent neuropsychological task performance and (4) any current drug abuse. Healthy volunteers were recruited as controls and had not prior history of psychiatric disease, mental retardation, neurological or general medical illnesses, including substance dependence, as determined by using an abbreviated version of the Comprehensive Assessment of Symptoms and History (CASH) [22]. We recruited control subjects according to specific socio-demographical characteristics based on age, gender, handedness and years of education. All participants provided informed consent for participation in the study. The research was approved by the hospital's Ethics Committee.

### Assessments

#### Psychopathology

Psychiatric symptoms were assessed by trained raters with the 24-item Brief Psychiatric Rating Scale (BPRS) [23]. The BPRS is a scale which evaluated symptoms reported by the patient (items 1–14 where items 7, 12 and 13 valuating also the behaviour of the patient observed during the interview) and signs identified during the interview (items 15–24). The attribution of the score considers severity, frequency and functional impairment (score increasing from 1 to 7, with the possibility to specify if an item has not been evaluated: NV). The scale includes the affective-anxious dimension—resulted from the Excitement subfactors (BPRS_Exc: items 6, 7, 12, 13 and 20–24) and Anxiety/Depression subfactors (BPRS_Anx/D: items 1–5 and 19) and the more specifically psychotic symptoms Negative Symptoms subfactors (BPRS_NS: items 13, 14, 16, 17, 18, 20, 24) and Positive Symptoms subfactors (BPRS_PS: items 8–11 and 15) [24].

#### Suicidal dimension

The Columbia-Suicide Severity rating Scale (C-SSRS [25];) is a semi-structured clinical interview that assesses suicidal dimension exploring four different areas: severity of suicidal ideation (1 = wish to be dead, 2 = not specific active suicidal thoughts, 3 = suicidal thoughts with methods, 4 = suicidal intent and 5 = suicidal intent with plan); intensity of suicidal ideation (five items each rated 0 to 5: frequency, duration, controllability, deterrents, and reasons); suicidal behaviour (concrete attempts, failed attempts, interrupted attempts and preparatory acts); actual or potential lethality of suicidal behaviours [25].

#### Global functioning

The Global Assessment of Functioning (GAF) [26] is a scale used to assess patient’s global functioning. The clinician attributes a score from 0 to 100 (there are 10 sub-intervals) to social and role functioning.

#### Neurocognitive assessments

The MATRICS Consensus Cognitive Battery (MCCB) [11] was developed to establish a standardised method to measure cognitive function to stimulate the development of new drugs for the cognitive deficits of schizophrenia The MCCB was also used to evaluate neurocognitive functions in other mental illness [27]. It comprises 10 neuropsychological tests (Category Fluency – Animal Naming; Brief Assessment of Cognition in Schizophrenia Symbol Coding; Trail Making Test – Part A; Continuous Performance Test – Identical Pairs; Wechsler Memory Scale Spatial Span; Letter-Number Span; Hopkins Verbal Learning Test – Revised; Brief Visuospatial Memory Test – Revised; Neuropsychological Assessment Battery – Mazes; Mayer-Salovey-Caruso Emotional Intelligence Test). The battery assesses seven cognitive domains including speed of processing, attention and vigilance, verbal learning, working memory, reasoning and problem solving, visual learning and social cognition [28].

### Statistical analysis

The socio-demographic and clinical characteristics of the diagnostic groups were compared using the chi-square test (χ2) for nominal variables. Unidirectional analysis of variance (ANOVA) was used for continuous variables. The α (alpha) level for these tests was set for *p* = 0.05.

A Multivariate Analysis of Variance (MANOVA) were conducted for each cognitive domain using the suicide size criteria as independent variables (SA vs SI vs PC vs HC). Once the significance of the initial model was verified (Wilk's Lambda value = 0; sig. = 0), we performed the Fisher Least Significant Difference (LSD) post-hoc test to identify the pairs of averages with a value statistically significant. We also conducted the Multivariate Covariance Analysis (MANCOVA) on the neurocognitive domains with the independent variables as covariates (age, education, duration of illness, diagnosis, BPRS_Tot; BPRS_Ex; BPRS_Anx-D; BPRS_Neg; BPRS_Pos), to further exclude their possible confounding effect, which may impact both cognitive performance and risk for suicidal behavior. Statistical analyzes were performed using SPSS software version 23.

## Results

The socio-demographic and clinical characteristics in the different groups are described in Table 1. The mean age of the subjects was 39.7 (SD = 12.6; *p* = 0.8). The sample did not differ by gender (χ^2^ = 3.1; df = 3; *p* = 0.3), diagnosis (χ^2^ = 8.1; df = 4; *p* = 0.08); years of education (mean = 12.8; SD = 3.3; p = 0.8), duration of illness (mean = 17; SD = 11.9; *p* = 0.7). The scores of the GAF and BPRS_Total did not differ among clinical groups (GAF: Mean = 52; SD = 8.5; *p* = 0.3; BPRS_Tot: Mean = 47.3; SD = 11.8; *p* = 0.7).

**Table 1:** Socio-demographic and psychopathological characteristics of the sample

	Patient Controls (with no Suicidal ideation/behaviour) (*N* = 27)			Patients with Suicidal Ideation (*N* = 20)			Patients with Suicide Attempts (*N* = 23)			Healthy Controls (*N* = 36)			Analysis		
	Mean	SD	95% CI	Mean	SD	95% CI	Mean	SD	95% CI	Mean	SD	95% CI	F	Df	p
Age	41.1	12.6	36.2–45.9	38.3	13.8	32.6–44	38.3	13.0	33–43.5	40.4	12.1	36.2–44.6	0.3	3	0.8
Years of education	13.1	1.9	11.8–14.4	13	2.5	11.4–14.5	13.1	3.4	11.7–14.5	12.4	4.3	11.3–13.5	0.2	3	0.8
Duration of illness (yrs)	17.3	11.4	13.6–21.1	15.55	12.7	11.1–19.9	18	12.3	13.9–22				0.2	2	0.7
GAF	53.4		50.8–56.1	52.2		49.1–55.3	50.1		47.2–52.9				0.9	2	0.3
BPRS Total	45.9		42.2–49.6	48.1		43.8–53.4	48.1		44.1–52.1				0.2	2	0.7
	Male	Female	Analysis												
			χ2	Df	p										
N (%)	61 (57%)	45 (43%)	3.1	3	0.3										

**Fig. 1:**
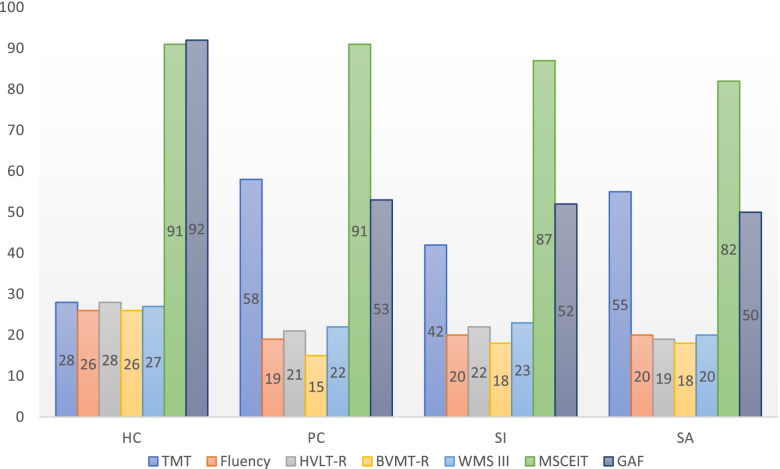
Neuropsychological profiles in different suicidal risk groups: significant comparisons (Post Hoc Analysis): TMT: HC < PC, HC < SA (the lower the score, the highest the performance). HVLT-R: HC > PC, HC > SI, HC > SA. Fluency: HC > PC, HC > SI, HC > SA. MSCEIT: HC > SI, HC > SA; PC > SI, PC > SA; SI > SA. GAF: HC > PC, HC > SI, HC > SA. *TMT* Trail Making Test, *FLUENCY *Animal Naming, *HVLT-R* Hopkins Verbal Learning Test – Revised, *BVMT-R* Brief Visuospatial Memory Test, *WMS III* Wechsler Memory Scale Spatial Span, *MSCEIT* Mayer-Salovey-Caruso Emotional Intelligence Test, *GAF* Global Assessment of Functioning, *HC* healthy control group, *PC* patient control, *SI* suicidal ideation, *SA* suicide attempt

Taken all together post-hoc comparisons showed that clinical groups showed lower scores than the healthy group for most MATRICS tests. Six out of ten tests showed significant differences between groups: Trail Making Test (TMT), FLUENCY, Hopkins Verbal Learning Test – Revised (HVLT-R) Brief Visuospatial Memory Test (BVMT-R) Wechsler Memory Scale (WMS III) and Mayer-Salovey-Caruso Emotional Intelligence Test (MSCEIT). In all tests, HC performed better than the other groups. After applying the Bonferroni correction with *p* < 0.001, only the domain of social cognition (MSCEIT) differentiated the three clinical subgroups one from each other (Table 2, Fig. 1).

**Table 2:** Comparison among groups (MANCOVA analysis, adjusted for possible confounding variables (age, sex, years of education, duration of the illness, diagnosis, BPRS Total and subfactors)

Cognitive domain	Cognitive task	HC Mean (SD)	PC Mean (SD)	SI Mean (SD)	SA Mean (SD)	F	Df1	Df2	P	Significant post-hoc
Speed of processing	TMT	28.7 12.1	58.0 34.9	41.8 16.7	55.1 19.4	2.177	3	102	**.041**	HC < PC, HC < SA
	BACS	56.8 12.9	37.9 13.3	42.0 13.3	35.8 12.2	1.141	3	102	.337	
	FLUENCY	25.8 6.3	18.9 5.2	20.2 4.8	19.7 6.8	9.955	3	102	.**000**	HC > PC, HC > SI, HC > SA
Attention	CPT	2.8 0.8	2.5 .9	2.4 1.1	2.3 0.9	0.489	3	102	0.691	
Verbal learning	HVLT-R	26.4 4.3	21.7 6.7	23.2 2.8	20.2 5.2	0.353	3	102	**.047**	HC > PC, HC > SI, HC > SA
Visual learning	BVMT-R	25.8 6.7	15.3 7.0	18.0 5.8	18.4 9.4	1.427	3	102	**.050**	HC > PC, HC > SI, HC > SA
Working memory	WMS III	26.4 4.3	21.7 6.7	23.2 2.8	20.2 5.2	0.994	3	102	**.039**	HC > PC, HC > SA
	LNS	16.2 2.8	12.3 4.0	13.3 3.7	12.0 3.7	0.499	3	102	.684	
Reasoning and Problem solving	NAB	18.6 6.3	9.6 6.6	13.6 7.3	10.6 6.7	0.335	3	102	.800	
Social cognition	MSCEIT	91.1 9.7	91.2 9.3	86.5 10.6	82.3 7.4	10.549	3	102	**.000**	HC > SI, HC > SA, PC > SI, PC > SA, SI > SA
Global functioning	GAF	92.1 1.2	53.4 1.3	52.2 1.5	50.1 1.4	23.900	3	102	**.000**	HC > PC, HC > SI, HC > SA

*TMT* Trail Making Test, *BACS* Brief Assessment of Cognition (Symbol-Coding), *FLUENCY* Animal Naming, *CPT* Continuous Performance Test – Identical Pairs, *HVLT-R* Hopkins Verbal Learning Test – Revised, *BVMT-R* Brief Visuospatial Memory Test, *WMS III* Wechsler Memory Scale Spatial Span, *LNS* Letter-Number Span, *NAB* Neuropsychological Assessment Battery, *MSCEIT* Mayer-Salovey-Caruso Emotional Intelligence Test, *GAF* Global Assessment of Functioning, *HC* Healthy control group, *PC* patient controls, *SI* Suicidal ideation, *SA* suicide attempt

## Conclusions

In the present study, we aimed to analyze differences in neurocognitive profiles among patients with established mental illness but at different degrees of suicide risk. In order to exclude the possible confounding effect of symptoms on suicidality, we adjusted our results for psychopathological variables (excitement, anxiety/depression, negative symptoms, and positive symptoms) and socio-demographic characteristics (age, years of illness).

First, we found that our clinical sample performed worse than the healthy control group in almost all domains.

In particular, we found that:

1. Globally, the group of patients performed worse than the healthy control group on all domains; consistently the global functioning was significantly higher in HC compared to the group of patients;
2. Compared to HC, SI and SA groups performed significantly worse on social cognition task**,** whereas there were no significant differences between PC and HC;
3. SA performed worse than SI on social cognition task.

The first finding is in line with current literature that shows strong evidence for a moderate neurocognitive impairment across domains in psychiatric population compared to healthy controls [29–32], regardless of the presence of suicide risk.

The second finding suggests that social cognition impairment differentiated suicide risk groups from both HC and psychiatric patients without suicide ideation or attempts.

The third finding showed that social cognition is more impaired in SA than in patients who only have thoughts of suicide, thus, as we hypothesized, different degrees of suicide risk also correspond to different stage of social cognition impairment.

Given that it is already well-known that social cognition impairment is a crucial suicide risk factor in schizophrenia and other psychotic disorders [33, 34]), the novelty of our findings is that social cognition is involved in suicidal risk per se, independently from diagnostic categories. In fact, other cognitive domains explored in this study did not differentiated clinical groups from HC, suggesting that all other neurocognitive deficits may be related to comorbid mental disorders and that they may contribute to the suicide risk without differentiating a patient with or without suicide ideation or suicide attempt history. On the other hand, the domain of social cognition is involved in both suicidal ideation and suicide attempts, thus playing a crucial role in suicidality among patients diagnosed with serious mental illness. Social cognition is an important context-related mediator in shaping representations of oneself and others; it underlies the mental operations guiding social behavior and revolves around concepts such as attribution, intention, and agency. To ensure efficient social interaction, people have to distinguish between self and others and integrate other people’s thoughts, emotions, and behavioral intentions with their own. Indeed, social cognition encompasses a large set of cognition functions and processes, such as expression, recognition and regulation of emotions, theory of mind, which is the ability to infer and internally represent the mental states of others, and hence to attribute and interpret desires, beliefs, intentions and thoughts as determinants and predictors of behavior [35].

Notably, in the present study, the assessment of social cognition was carried out through the Mayer-Salovey-Caruso Emotional Intelligence Test (MSCEIT) “Managing Emotion” (ME) section, that is considered a key measure for social cognition, although it was originally conceived as an emotional intelligence measure. It is a well-documented psychometric test with an important validation background for several languages [36]. Emotional intelligence (EI) was first described and conceptualized by Salovey and Mayer [37] as an ability-based construct analogous to general intelligence. They argued that individuals with a high level of EI had certain skills related to the evaluation and regulation of emotions and that consequently they were able to regulate emotions in themselves and in others in order to achieve a variety of adaptive outcomes. Unlike many assessments of emotion processing, the MSCEIT-ME branch is performance-based as participants are prompted to solve emotional problems rather than report their emotional abilities. The task includes two subtests assessing social and emotion management. A total of eight brief vignettes describing problematic social situations are read aloud to the participant, followed by four different ways a person might react to the situation. The choices reflect varying degrees of emotional reactivity, with some clearly being more adaptive than others. For example, one vignette describes being cut off in traffic while driving and gives possible actions one might take, including: a) getting revenge by then cutting the other driver off, b) continuing to drive, c) screaming at the driver, and d) never driving there again. The participant is then asked to rate the effectiveness of each of the four actions using a five-point scale ranging from very ineffective to very effective.

Given that MSCEIT enables the evaluation of emotion processing similar to the evaluation of general intelligence by neurocognitive assessments, its Managing Emotions (ME) subscale was recommended by the NIMH- MATRICS (National Institutes of Mental Health, Measurement and Treatment Research to Improve Cognition in Schizophrenia) committee as a measure of social cognition in schizophrenia in the widely implemented MATRICS Consensus Cognitive Battery (MCCB) [11, 12].

Finally, social cognition represents the ability to not only monitor, recognize, and reason about one’s own and other people’s emotions, but also to use this emotional information to guide one’s thinking and actions. This explains why social cognition is more compromised in attempters than in patients with only thoughts of suicide. Accordingly, a recent meta-analysis confirmed that a high level of EI plays an important role in protecting against suicidal behavior [38].

Interestingly, recent neuroimaging studies confirm the involvement of social cognition in suicide risk. In neurobiological terms, areas like the right orbitofrontal cortex and the right anterior cingulate cortex, involved in social cognition and discriminating between positive and negative emotions, seem to play a key role in self-regulating affect and thoughts, and could be an important factor in order to understand suicidal ideation [39, 40]. Notably, patients with a history of suicidal behavior showed different responses to negative emotional cues compared to well-matched patients with low suicidal risk in several regions of the prefrontal cortex, and in particular increased activity in the right lateral orbitofrontal cortex (Brodmann’s area 47) and decreased activity in the right superior frontal gyrus (area 6) [41]. Brodmann’s area 47 has been implicated in the processing of facial expressions and may be especially responsive to subjectively salient or mood-congruent expressions. Indeed, a functional neuroimaging study [42] showed a different activation in left posterior insula and supramarginal gyrus compared with both psychiatric without a history of suicidal behaviour and healthy controls. Thus, both right orbitofrontal cortex and left insula that are crucial areas for social cognition and may be involved in suicidal risk.

The present study has some limitations that should be consider. First, due to the small sample size, the risk of biased findings is increased. We recruited participants among real-world patients, and thus selection bias cannot be ruled out. On the other hand, a real-life observational study design allows obtaining data that are applicable in daily clinical practice. Second, the cross-sectional design does not allow formulation of specific hypotheses regarding the causal role or the interplay between variables explored along the course of the illnesses, longitudinal studies investigating patients at multiple stages of the disease process, will be better equipped to test the interplay between neurocognitive factors and suicide risk. Third, the patient population is not drug free and we do not know in how much this may affect interpretation of data.

Overall, the present study indicates that clinicians have to assess carefully neurocognitive profiles when evaluating suicide risk in psychiatric illness, regardless of diagnosis. This is important for two reasons; the first is the prevention of possible suicide behaviors in the presence of history of suicide attempts and social cognition impairment; the second reason is of a therapeutic nature; in fact, implementing social cognition may be a new therapeutic approach to reduce suicide risk.

Cognitive deficits in psychiatric patients are already important therapeutic targets of intervention [17]. Despite the paucity of intervention studies considering cognition as a therapeutic target in suicide patients, training self-regulatory processes, including decision-making skills, efficient problem solving, and impulse control present a likely potential for clinical use in suicide prevention [43]. Behavioral and cognitive interventions has been associated with reductions on suicide ideation, probably by targeting different cognitive dysfunctions associated with suicide behaviors, in addition to anxiety and depressive symptoms [44]. Thus, specific interventions toward these cognitive domains, such as attentional bias, impulsivity, problem solving, and decision-making, could help to maximize the efficacy of the available therapeutic options. Scientific evidence concerning the possibility of ameliorating cognitive functioning in patients with severe mental illness is still limited, but researchers are making advances in this area to ultimately achieve this goal [45].

Finally, we intend to promote a trans-nosographic and trans-staging view of suicidal risk and our results suggest that social cognition may represent a clinical marker for defining the stage of severity of suicidal risk. Further research may offer relevant information on this, with the extension to affect regulation and decision-making functions being other key factors explaining self-injurious behaviors [46], not assessed in the present study.

## Data Availability

The measures section provides references to where all materials used in this article can be obtained from. The analysis script for the present research can be obtained from the authors upon request.
